# Population analysis of microsatellite genotypes reveals a signature associated with ovarian cancer

**DOI:** 10.18632/oncotarget.2933

**Published:** 2015-03-04

**Authors:** Natalie C. Fonville, Zalman Vaksman, Lauren J. McIver, Harold R. Garner

**Affiliations:** ^1^ Virginia Bioinformatics Institute, Virginia Tech, Blacksburg, VA 24061, USA

**Keywords:** Ovarian Cancer, Biomarkers, The Cancer Genome Atlas, Breast Cancer, 1,000 Genomes Project

## Abstract

Ovarian cancer (OV) ranks fifth in cancer deaths among women, yet there remain few informative biomarkers for this disease. Microsatellites are repetitive genomic regions which we hypothesize could be a source of novel biomarkers for OV and have traditionally been under-appreciated relative to Single Nucleotide Polymorphisms (SNPs). In this study, we explore microsatellite variation as a potential novel source of genomic variation associated with OV. Exomes from 305 OV patient germline samples and 54 tumors, sequenced as part of The Cancer Genome Atlas, were analyzed for microsatellite variation and compared to healthy females sequenced as part of the 1,000 Genomes Project. We identified a subset of 60 microsatellite loci with genotypes that varied significantly between the OV and healthy female populations. Using these loci as a signature set, we classified germline genomes as ‘at risk’ for OV with a sensitivity of 90.1% and a specificity of 87.6%. Cross-analysis with a similar set of breast cancer associated loci identified individuals ‘at risk’ for both diseases. This study revealed a genotype-based microsatellite signature present in the germlines of individuals diagnosed with OV, and provides the basis for a potential novel risk assessment diagnostic for OV and new personal genomics targets in tumors.

The American cancer society estimated there would be 21,980 new cases of ovarian cancer (OV) and 14,270 deaths in 2014 [1] with epithelial ovarian carcinoma accounting for approximately 90% of all cases [2], making ovarian cancer the most lethal gynecological cancer in the United States [3], and the fifth most deadly cancer in women. The 5-year survival rate is approximately 50% because early diagnosis is not usually possible as symptoms of this cancer are nonspecific and common testing methods are not likely to detect this disease in the early stages [4].

Recently there have been an emergence of large-scale -omics projects whose goal is to allow accurate and complete analysis of the mutational profile of disease, and the availability of this data has already yielded novel insights into the mutational spectrum of several cancers, including ovarian cancer [5, 6]. However, the analysis of genomic variation is not yet complete as there remains a largely overlooked source of variation at certain genomic regions such as microsatellites. Microsatellites are low complexity, repetitive DNA regions that have been associated with morphological changes and human diseases, notably with triplet repeat instability disorders [7], and have traditionally been thought of as having extremely high levels of polymorphism and heterozygosity, compared to high complexity DNA sequences [8]. However, our analysis of all available loci has shown that 98% of microsatellite loci are invariant, that is have less than a 1% polymorphism rate (manuscript in preparation). Microsatellites are ubiquitous and are over-represented in the human genome compared to expected levels that would be present by chance [9]. Their high frequency and relative invariance in disease-free populations make microsatellites good candidates to become informative markers for cancer and disease progression. Recently, algorithms specifically designed to accurately assess variation within microsatellites have been developed [10–13]. The growing availability of high-quality next-generation sequencing data, our accurate microsatellite genotyping algorithm and the ability to do genomics on a population scale have now combined to allow us to analyze microsatellite variation en masse. We have performed an analysis of microsatellites on a population level to (A) determine the normal range of variation, defined as variation found in individuals sequenced as part of the 1000 Genomes Project (1kGP), (B) use the genotype distribution at each microsatellite locus among healthy individuals as the baseline for comparison to genomes from individuals diagnosed with epithelial ovarian cancer from The Cancer Genome Atlas (TCGA) to assess the ability of greater OV-associated microsatellite variation as a potential novel risk assessment method and (C) use matching tumor and germline genotype distributions to identify “hot spots” for acquired variation in tumors as a potential personal genomics tool.

## RESULTS

### Establishing the normal range of microsatellite variation from the 1kGP

In order to evaluate microsatellite instability in cancer patient data from TCGA, we first needed to establish a baseline for variation within the healthy population at each microsatellite locus. To do this we analyzed variation at each microsatellite locus in 249 females of European ancestry from the 1kGP data set ethnically matched to the OV cancer population of The Cancer Genome Atlas (TCGA). As our control set was from the 1,000 genomes project, no medical or phenotype information was provided for these individuals. At the time of sequencing, this population consisted of people who had not been diagnosed with cancer, therefore it should not be enriched for cancer-associated variants; however, given that a woman's lifetime risk of developing ovarian cancer is 1:72, we would expect that approximately 3 individuals in the healthy cohort will develop OV.

For comparisons to the OV data set, data from 249 females of European ancestry sequenced by the 1kGP was used to determine baseline variation. This control population was originally used for a similar study to identify a set of microsatellites that could distinguish Breast Cancer germline samples from the healthy population [14]. The same control group is used in this study to allow comparisons between the two signature microsatellite sets.

### Microsatellite variation in OV

Next-generation sequencing data from 305 germline samples from females diagnosed with OV, were obtained from TCGA [15]. The genotype at all microsatellite loci with at least 15X read depth coverage was determined. We then identified the most common genotype present within the healthy population and designated it as the ‘modal’ genotype. All other genotypes are then considered non-modal. For each population then we determined the frequency of modal and non-modal genotypes at each locus. Comparison of this frequency between the healthy and OV populations led to identification of 60 statistically significant microsatellite loci (Table 1) that passed stringent Type 1 and False Discovery tests (*p* < 0.001 by Fisher's exact test and adjusted *p* < 0.01 by Benjamani Hochberg).

**Table 1:** Statistically significant loci that differentiate healthy from OV cancer germlines Loci demarked in bold were also informative for breast cancer using a similar approach. Encode and other element designations (from the UCSC browser) are as follows: 1 – Transcription factor binding site, 2 – DNaseI hypersensitivity locus, 3 – Spliced EST, 4 – H3K27Ac mark (found near active regulator elements), 5 – human mRNA.

Microsatellite Locus	Motif	Region	Gene	Encode element / other	1kGP samples genotyped	Percent 1kGP Non-modal	OV Samples Genotyped	Percent OV Non-modal	Relative Risk
chr5:122714135–122714152	A	intron	CEP120		50	10%	70	64%	6.4
chr2:91886031–91886042	A	intergenic	–		186	14%	240	48%	3.4
**chr5:133944044–133944059**	**T**	**intron**	**SAR1B**		**17**	**29%**	**13**	**100%**	**3.4**
chr5:158511580–158511594	A	intron	EBF1	1	33	24%	38	82%	3.4
chr10:69699479–69699497	AT	intron	HERC4		79	14%	120	39%	2.8
chr2:223339530–223339550	T	intron	SGPP2		22	36%	41	85%	2.3
chr7:81695843–81695858	A	intron	CACNA2D1		42	38%	83	87%	2.3
chr18:21120382–21120397	A	intron	NPC1	1, 2	42	43%	60	93%	2.2
chr13:49951024–49951057	ATAG	intron	CAB39L		140	26%	217	52%	2.0
chr2:234368716–234368729	A	intron	DGKD		20	50%	114	93%	1.9
chr11:30438959–30438973	T	intron	MPPED2		40	53%	87	14%	1.8
chr12:75901962–75901976	A	intron	KRR1		41	51%	94	12%	1.8
**chr9:5798652–5798666**	**A**	**intron**	**ERMP1**		**22**	**45%**	**91**	**2%**	**1.8**
**chr19:30106131–30106147**	**T**	**intron**	**POP4**		**30**	**53%**	**41**	**95%**	**1.8**
chr14:91928846–91928860	T	intron	SMEK1	2	28	54%	42	95%	1.8
chr1:149900986–149901001	A	exon	MTMR11	2, 3, 5	37	51%	54	89%	1.7
chr9:52626–52640	A	intergenic	-		44	55%	90	92%	1.7
**chr3:98299708–98299720**	**A**	**intron**	**CPOX**		**56**	**46%**	**42**	**10%**	**1.7**
chr20:44333327–44333340	T	intron	WFDC10B		80	45%	90	76%	1.7
chr4:186188374–186188387	A	intron	SNX25		75	47%	127	12%	1.7
**chr11:62565909–62565944**	**AA AAGA**	**intron**	**NXF1**		**37**	**38%**	**90**	**0%**	**1.6**
chr11:116691512–116691528	ACAG	exon	APOA4	5	156	53%	222	84%	1.6
**chr11:110128926–110128940**	**A**	**intron**	**RDX**	**3**	**42**	**40%**	**78**	**5%**	**1.6**
**chr17:63747018–63747031**	**A**	**intron**	**CEP112**	**1, 2, 3**	**48**	**40%**	**118**	**6%**	**1.6**
chr9:133498230–133498244	A	intron	FUBP3		37	41%	83	8%	1.5
**chr6:49815874–49815887**	**T**	**intron**	**CRISP1**		**54**	**41%**	**108**	**11%**	**1.5**
chr8:121518869–121518882	T	intron	MTBP		39	36%	72	6%	1.5
chr12:22676634–22676648	A	intron	C2CD5		52	40%	97	12%	1.5
chr10:93579112–93579132	T	intron	TNKS2		43	37%	103	8%	1.5
chr17:47899281–47899294	A	intron	KAT7	3	30	33%	81	2%	1.5
chr3:50095097–50095118	T	intron	RBM6		61	36%	76	8%	1.4
chr7:36465607–36465621	T	intron	ANLN		90	44%	154	21%	1.4
chr19:21558016–21558032	TG	intron	ZNF738		159	48%	186	27%	1.4
chr12:106500161–106500174	A	intron	NUAK1	1, 2	53	32%	121	5%	1.4
chr17:57078816–57078830	A	intron	TRIM37	1, 4	33	27%	100	1%	1.4
chr1:169555368–169555380	A	intron	F5	1	82	28%	161	4%	1.3
**chr2:203680555–203680567**	**A**	**intron**	**ICA1L**		**99**	**24%**	**177**	**1%**	**1.3**
chr4:22444252–22444266	A	intron	GPR125		77	26%	111	4%	1.3
**chr20:5167156–5167168**	**T**	**intron**	**CDS2**	**2, 5**	**61**	**23%**	**146**	**0%**	**1.3**
chr17:66041872–66041885	T	intron	KPNA2	3	69	30%	159	10%	1.3
chr6:76728584–76728597	A	intron	IMPG1		68	24%	111	3%	1.3
chr10:22515002–22515024	A	intergenic	-		54	22%	111	2%	1.3
chr5:86679677–86679690	T	intron	RASA1	4	67	21%	116	1%	1.3
**chr15:89811883–89811895**	**T**	**intron**	**FANCI**		**47**	**19%**	**135**	**1%**	**1.2**
chr10:94266331–94266345	T	intron	IDE	2	82	18%	75	0%	1.2
chr18:2960513–2960525	A	intron	LPIN2	1,2,4	67	18%	90	0%	1.2
chr15:64972761–64972788	TG	intron	ZNF609		121	23%	208	8%	1.2
chr16:10783089–10783101	A	intron	TEKT5		66	17%	130	0%	1.2
chr4:71888333–71888347	T	intron	DCK	2	49	16%	111	0%	1.2
chr1:236721453–236721465	A	intron	HEATR1		101	17%	150	1%	1.2
chrX:11187894–11187905	T	intron	ARHGAP6		61	16%	187	2%	1.2
chr11:89534160–89534172	A	intron	TRIM49		80	15%	131	1%	1.2
chr6:89638989–89639003	A	intron	RNGTT		94	15%	130	1%	1.2
chr4:141448596–141448609	T	intron	ELMOD2		100	14%	157	1%	1.1
**chr6:170881390–170881402**	**T**	**3utrE**	**TBP**	**3,5**	**78**	**13%**	**221**	**0%**	**1.1**
**chr8:107704941–107704954**	**A**	**intron**	**OXR1**		**119**	**13%**	**188**	**0%**	**1.1**
chr7:31132236–31132248	T	intron	ADCYAP1R1	2	114	12%	192	2%	1.1
**chr8:30933817–30933828**	**T**	**intron**	**WRN**		**132**	**10%**	**230**	**0%**	**1.1**
**chr7:122757720–122757732**	**A**	**intron**	**SLC13A1**		**92**	**9%**	**183**	**0%**	**1.1**
chr19:20829219–20829233	AC	intron	ZNF626		203	5%	281	0%	1.1

Genotyping of microsatellites using methods that dramatically improve the accuracy of microsatellite allele calling [10], allowed us to evaluate microsatellite loci based on the genotype rather than haplotype. Sixty microsatellite loci were identified as significantly differentiating OV from healthy individuals (Table 1). Of these, only 13 (21.7%) had a modal genotype in the 1kGP that was either heterozygotic with only one of the two prominent alleles represented by the reference allele length or homozygotic and differed from the reference allele length for that microsatellite (Table 1). This substantiates our comparison based on the modal genotype within a population as being able to identify additional significant differences that may not have been identified using a single microsatellite allele length as a reference.

Three of the sixty informative microsatellite loci were exonic, and an additional locus has been identified in human mRNA (Table 1). One of the genes, MTMR11 (Entrez gene ID: 10903), is a member of the protein tyrosine phosphatase family, and has been shown to be downregulated in some HER2 breast cancers [16]. Another of the genes, APOA4 (Entrez gene ID: 337), has previously been identified as a potential biomarker for malignant tumor differentiation in OV [17]. The third exonic microsatellite was in the 3′UTR of TATA-binding protein TBP (Entrez gene ID: 6908). TBP and its associated factors (TAFs) make up transcription factor IID and coordinate transcription by RNA polymerase II. The 3′UTR is a common target for regulation by miRNA and therefore microsatellite variation in this region could potentially have effects on protein stability and, in the case of TBP, broader effects on cellular transcription.

Variation of intronic microsatellites has been shown previously to be capable of affecting mRNA splicing and may contribute to disease [18, 19]. Of the 60 informative microsatellite loci, 5 are associated with known spliced ESTs (Table 1). These include a microsatellite associated with KPNA2 (Entrez gene ID: 3838), a protein involved in nuclear transport and a potential regulator of DNA recombination and cell proliferation which has been shown to be upregulated in OV [20–22], and a microsatellite associated with KAT7 (Entrez gene ID: 11143), a lysine acetyltransferase that may act as a coactivator of TP53-dependent transcription [23].

Our analysis does not attempt to draw direct functional relationships between the OV-associated microsatellite genotypes and altered protein function, but functional annotation enrichment analysis of terms associated with the set of genes containing the 60 OV loci revealed enrichment of rRNA processing/ribosome biogenesis genes (*p* = 0.037). Ribosome biogenesis is a limiting factor that must be overcome in tumorigenesis [24] and therefore individuals with minor alterations in rRNA processing may be at increased risk of cancer.

### Risk classifier

**Figure 1:**
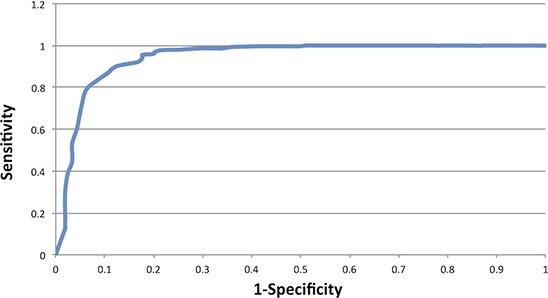
ROC curve using OV germline genotypes at the 60 microsatellite loci which had significantly different genotype distributions between OV and normal genomes

**Figure 2:**
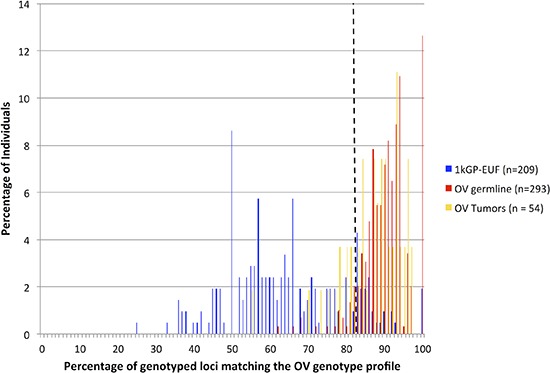
Microsatellite variation signature evaluated as a composite of the 60 statistically significant loci The non-overlapping distributions (healthy and cancer germline) is illustrative of the power to distinguish those populations. The dashed line marks where the 83% cut-off for calling a sample “OV-like” lies.

The presence of predominantly modal or non-modal genotypes at each of the 60 significant loci within the OV germline samples was used to create a ‘Cancer Profile’ for OV. We assembled a risk classifier based on the fraction of callable loci in each sample (healthy and cancer) for which the genotype matched the Cancer Profile. Based on the ROC curve (Figure 1) we determined the threshold for calling a germline genome as ‘cancer-like’ or having an OV-signature to be 83%. Therefore individuals having the cancer-associated microsatellite genotype at ≥ 84% of the signature microsatellite loci are classified by our method as ‘Cancer-like’ or potentially having an increased risk of OV. Using this cut-off, we classified the OV germline genomes as at risk for OV with a sensitivity of 90.1% and specificity of 87.6%. Excluding those samples in which less than 10% of the signature loci were genotyped, 264 of the 293 OV germline samples were identified as ‘cancer-like’ whereas only 26 of the 209 healthy females were flagged as having an increased risk for OV or ‘cancer-like’ (Figure 2). The 1kGP-EUF samples had a mean of 20.1 ± 8.8 of the 60 loci genotyped with 13.1± 7.4 identified as matching the cancer genotype whereas the OV germline samples had a mean of 25.0 ± 9.9 loci genotyped and 22.7 ± 9.0 loci identified as matching the cancer genotype (Table 2). This confirms that both populations were comparable in the per-exome mean number of loci genotyped, and that the difference lies in the number of loci that match the cancer profile.

**Table 2:** The mean numbers of OV and BC signature loci genotyped are within standard deviation for each population

Population	OV Loci GenotypedMean (SD)	OV “Cancer-like” LociMean (SD) / %	BC Loci GenotypedMean (SD)	BC “Cancer-like” LociMean (SD) / %
**1kGP-EUF**	20.1 (8.8)	13.1 (7.4) / 65%	15.5 (6.4)	8.9 (3.9) / 57%
**OV Germline**	25.0 (9.9)	22.7 (9.0) / 91%	16.5 (6.5)	13.4 (5.5) / 81%
**OV Tumor**	30.2 (6.7)	26.7 (6.9) / 88%	NA	NA
**BC Germline**	20.5 (7.7)	6.2 (7.2) / 79%	17.1 (4.9)	14.7 (4.3) / 86%

Although our signature loci were identified through comparison of the germline exomes of individuals diagnosed with OV to the control ‘healthy’ population, we found that when we analyzed the 54 OV tumor samples, 40 (74%) were classified as ‘cancer-like’ by our method, whereas only 14 (26%) were not identified as ‘cancer-like’ (Figure 2). Twenty-nine of the OV tumors were matched with germline samples. Table 2 shows that of these, both the tumor and germline were identified as ‘OV’ for 21 individuals (72%). There were an additional 7 individuals (33%) for which only the germline was identified as ‘OV’ and only one individual (4.8%) whose germline was not identified as ‘Cancer-like’ while the tumor sample was. There were no individuals for which neither the germline nor the tumor exome was classified as ‘Cancer-like’ by our method.

### Concordance of genotypes between the matched OV samples

We examined the matched germline and tumor samples in greater detail. We are able to genotype a similar number of microsatellites in the matched samples, with a mean of 33966 ± 3008 loci genotyped in the germline samples and a mean of 33881 ± 3621 in the tumor samples. A mean of 30013 ± 3042 loci were genotyped in both samples, and of those an average of 99.6% of those were concordant (had no change in genotype) between the two samples (Table 3). There was a mean of 135 loci per matched pair for which the genotype was discordant. However, we found that the discordance was primarily due to the tumor being homozygotic at a locus that was genotyped as heterozygotic in the germline (Table 4). Loss of heterozygosity (LOH) has been associated with OV [25], and the high percentage of discordant loci showing a loss of an allele is consistent with potential LOH.

**Table 3:** Concordance between genotype calls for those loci that were genotyped in matched tumor and germline samples

Participant ID (from CGHub)	Microsatellite loci genotyped in germline samples	Microsatellite loci genotyped in tumor samples	Microsatellite loci genotyped in both samples	Percent of loci whose genotype did not change	Total loci with a genotype change
**99f1ae02–86ec-4d93–8cd4–650bf6f02c10**	34520	35000	31644	99.56%	140
**4e6f88de-7624–4719-8234–4c9e5b2e2988**	36203	38368	32978	99.59%	134
**c0c3caab-9277–4a31-a96c-c607e38d5ccc**	37073	38394	32796	99.58%	138
**bc4bc342–20bf-40c3-af26–2c6f942da93d**	34085	33010	30611	99.54%	142
**d7f82e34–5b34–4e8c-a0cf-d7561bcea43c**	33219	34426	31315	99.72%	88
**15170c7f-5880–4fb6–82ce-68d3df0dfb68**	34699	32049	28937	99.55%	130
**1d192835–524e-429d-bf74–3c4727acb446**	26236	30068	24182	99.57%	103
**067c5c61-d147–4b08-ab8a-32c30969d564**	32870	32752	29467	99.59%	120
**fe402983–70da-44db-b7b1-c32702ddde26**	33095	34636	30829	99.55%	139
**25a0a9e6–4f5b-45d8–8f34-abfd31d5ff1b**	28086	30409	26132	99.53%	123
**94bd4c68–4bfc-4db3–9365-97c867747133**	38903	35810	33186	99.44%	185
**538acb2a-c4ca-4656-a91c-841a42dbf15f**	30982	30940	28150	99.59%	116
**9bf16a89–2fc7–4c08–93bc-3105eec5c3cc**	36652	35583	30508	99.50%	153
**f007fa7a-7da9–4cb0–8aea-623af1a122c5**	37568	39147	33420	99.49%	172
**bc3e0b74-ea09–46a5–9f61–16bd15ffd883**	28946	38352	27525	99.30%	192
**44493c23–82e9–4d9f-8e3c-7b3f9ae44970**	31361	32433	28154	99.53%	132
**700e91bb-d675–41b2-bbbd-935767c7b447**	32455	31604	29193	99.62%	111
**8783e4b0–2b62–45d5–8cd9-f5a71cc0138e**	33192	33002	29848	99.63%	109
**d0673efd-3315–4dd5–8ab6–912bfa07dceb**	32512	33874	29947	99.45%	166
**60cce7ac-d27d-44a6–9873-ecf91da5e906**	35536	33135	30532	99.50%	153
**a85f6f9c-1e1d-44fc-85eb-3b2d96cfbc61**	34736	34209	31701	99.56%	138
**66dc6379-a98b-498f-8109-e3a811d043ea**	38597	37074	33637	99.57%	144
**ee0a4a13–613e-4c5d-96c3–8083a013702d**	33755	35583	31398	99.61%	122
**a88b7e66–5f12–4023-a7e2-fcfbd1f25977**	33402	30381	28370	99.51%	138
**cbc5b936-ead5–4858-ab90-e639402789b0**	38030	35882	33150	99.52%	158
**7248cd60-be22–44bc-bc58-f644db0940a2**	36368	20670	19339	99.81%	36
**14c58def-60ee-48e0-a74b-da4eb77ef344**	33007	33671	30530	99.61%	119
**8a6d2ce3-cc57–451b-9b07–8263782aa23f**	33456	34118	30483	99.48%	160
**4d71dd15-cd01–4dae-ad70–6dc325140207**	35475	37981	32405	99.53%	151

**Table 4:** Microsatellite loci whose genotypes between matched tumor and germline samples were discordant predominantly showed loss of an allele

Participant ID (from CGHub)	Total Number of discordant loci	Percent of discordant loci with LOH	Percent of discordant loci with an allele gain	Percent of discordant loci with no concordant allele
**99f1ae02–86ec-4d93–8cd4–650bf6f02c10**	140	78%	21%	1%
**4e6f88de-7624–4719-8234–4c9e5b2e2988**	134	72%	25%	2%
**c0c3caab-9277–4a31-a96c-c607e38d5ccc**	138	65%	28%	7%
**bc4bc342–20bf-40c3-af26–2c6f942da93d**	142	80%	15%	4%
**d7f82e34–5b34–4e8c-a0cf-d7561bcea43c**	88	39%	60%	1%
**15170c7f-5880–4fb6–82ce-68d3df0dfb68**	130	75%	19%	5%
**1d192835–524e-429d-bf74–3c4727acb446**	103	79%	17%	5%
**067c5c61-d147–4b08-ab8a-32c30969d564**	120	69%	28%	3%
**fe402983–70da-44db-b7b1-c32702ddde26**	139	64%	30%	6%
**25a0a9e6–4f5b-45d8–8f34-abfd31d5ff1b**	123	66%	32%	2%
**94bd4c68–4bfc-4db3–9365-97c867747133**	185	71%	28%	1%
**538acb2a-c4ca-4656-a91c-841a42dbf15f**	116	72%	27%	1%
**9bf16a89–2fc7–4c08–93bc-3105eec5c3cc**	153	69%	29%	3%
**f007fa7a-7da9–4cb0–8aea-623af1a122c5**	172	71%	27%	2%
**bc3e0b74-ea09–46a5–9f61–16bd15ffd883**	192	82%	13%	6%
**44493c23–82e9–4d9f-8e3c-7b3f9ae44970**	132	70%	27%	3%
**700e91bb-d675–41b2-bbbd-935767c7b447**	111	66%	32%	2%
**8783e4b0–2b62–45d5–8cd9-f5a71cc0138e**	109	73%	22%	5%
**d0673efd-3315–4dd5–8ab6–912bfa07dceb**	166	73%	23%	4%
**60cce7ac-d27d-44a6–9873-ecf91da5e906**	153	73%	25%	3%
**a85f6f9c-1e1d-44fc-85eb-3b2d96cfbc61**	138	67%	29%	4%
**66dc6379-a98b-498f-8109-e3a811d043ea**	144	69%	28%	3%
**ee0a4a13–613e-4c5d-96c3–8083a013702d**	122	67%	30%	2%
**a88b7e66–5f12–4023-a7e2-fcfbd1f25977**	138	81%	16%	3%
**cbc5b936-ead5–4858-ab90-e639402789b0**	158	72%	25%	3%
**7248cd60-be22–44bc-bc58-f644db0940a2**	36	83%	14%	3%
**14c58def-60ee-48e0-a74b-da4eb77ef344**	119	80%	19%	1%
**8a6d2ce3-cc57–451b-9b07–8263782aa23f**	160	83%	15%	3%
**4d71dd15-cd01–4dae-ad70–6dc325140207**	151	64%	32%	4%

### Cross-analysis with BC

**Figure 3:**
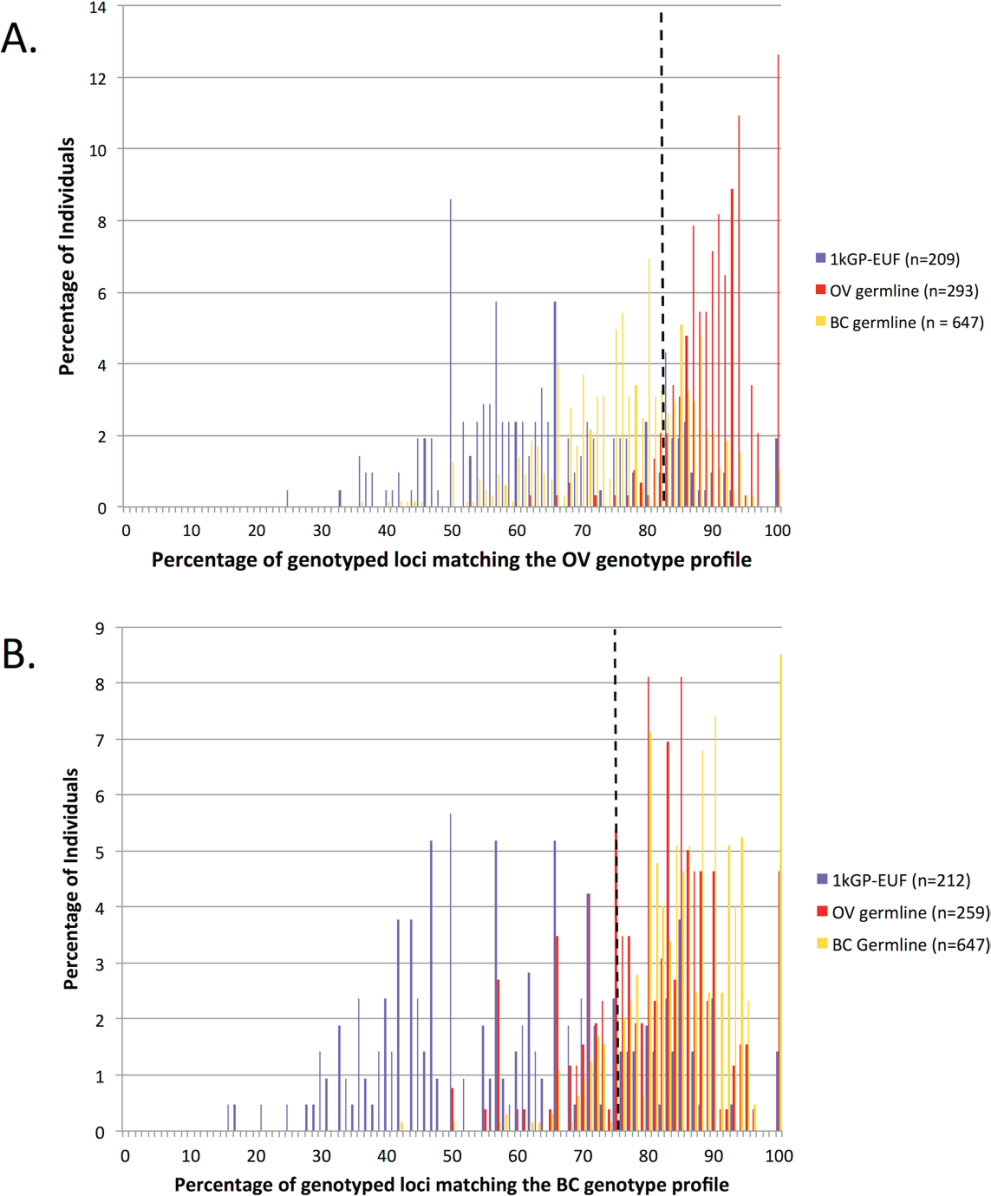
Cross analysis of the OV and BC samples and significant loci sets **(A)** Evaluation of the 1kGP-EUF healthy control, OV and BC germline exomes using the OV-signature set of microsatellites. **(B)** Evaluation of the 1kGP-EUF healthy control, OV and BC germline exomes at the BC-signature set of microsatellites.

The link between OV and breast cancer (BC) is well documented [26], however most of the studies have focused on hereditary BC/OV which can be attributed to BRCA1/2 [27]. There may also be some overlap in risk between non-hereditary BC and OV. We examined the overlap in the loci identified in this study as markers for OV risk and those identified in a similar study of BC individuals [14]. Fifteen of the 60 OV-associated loci were also identified as significant between BC and healthy individuals (demarked with blue, Table 1). We analyzed 647 BC germline samples obtained from TCGA using the OV profile and found that 193 (30%) of the BC individuals fall above the 83% cut-off of loci match the OV profile and were therefore classified by our method as ‘cancer-like’ for OV (Figure 3A). The overlap seen here in both the 15 loci that were included in both cancer-signature sets and the individuals that were classified has ‘cancer-like’ for both the signatures suggests that the link between BC and OV carries through in our method. In the reciprocal study, we analyzed each of the OV germlines at the published BC loci [14] and found that 181 (70%) of the 259 OV individuals were also classified as ‘cancer-like’ for BC as compared to 564 (87%) of BC individuals classified as ‘cancer-like’ using the BC signature (Figure 3B). Of the 259 OV exomes that could be evaluated by both signatures, 166 (64.1%) were classified as ‘cancer-like’ using both the BC and OV signature loci sets while 66 (25.5%) were classified as ‘cancer-like’ by just the OV signature set and 15 (6.8%) by just the BC set. Only twelve individuals were not classified as ‘cancer-like’ using either signature. Conversely, of the 190 1kGP-EUF exomes that were evaluated by both signatures, 11 (5.8%) were identified as cancer-like by both signatures whereas 135 (71%) were not identified as cancer-like by either signature.

## DISCUSSION

Currently there are few biomarkers for early detection of OV, and our evaluation of an OV-signature of microsatellite variation could prove to be a valuable additional resource for identifying those individuals who would benefit from increased surveillance for OV. Our analysis of microsatellites from OV genomes from TCGA is unique in that it not only assesses genomic microsatellite variations that arise in tumors, which are well known to be unstable, but it can identify low, but significant, levels of genomic microsatellite variation within the germline compared to the general population. We were able to identify a distinct subset of 60 microsatellite loci associated with OV, each of which has power to differentiate the germlines of healthy females from those that have developed ovarian cancer. Individually, these also inform as to possible mechanism and are potential new therapeutic targets, but together as a set, they could be used to identify genomes that carry an ‘OV risk signature’. The most significant finding is that we were able to identify the OV signature in the germline of OV patients, not the tumor, therefore, variation at these loci has potential use as a risk-assessment screening method and may be included along with other analyses in informing a physician's decision on patient care and monitoring of an individual. The specificity of this assay is too low to be used as a general population screen, so would be most appropriately applied to the subset of women who have a family history of breast or ovarian cancer or other risk factors such as unexplained infertility [28, 29]. In addition to analyzing OV germline exomes at the 60 signature loci, we were able to perform a cross-analysis of OV and BC using the OV loci found here and 55 loci that were previously published as differentiating BC from normal. Fifteen loci were joint loci, i.e. were identified in both risk sets including in genes that have roles in DNA repair (e.g. WRN and FANCI) or roles in transcription regulation (TBP). The overlap in informative loci found in both OV and BC may represent those loci that increase broad-spectrum cancer risk. In addition, we were able to identify 30% of breast cancer exomes as ‘at risk’ for OV whereas 70% of OV were classified as ‘at risk’ for BC. This may indicate enhanced susceptibility to a second primary tumor development in these patients. As more genomic data becomes available it will be critical to validate these observations, and determine how these variants imply mechanism, as part of translating these findings into clinical utility.

## METHODS

### Data sets

A set of 249 exomes from healthy European females was used as the control group to establish the expected microsatellite genotypes. These individuals were exome sequenced at high coverage by the 1000 Genomes Project [30]. These were compared to exome sequencing data from 305 germline samples from individuals with ovarian cancer (OV) and 54 tumor samples (29 of which were matched), which were sequenced by The Cancer Genome Atlas for study phs000178.v5.p5 [6]. Because of the documented assembly inaccuracies at microsatellite loci for all the data emerging from all nextgen sequencing projects, we did not use the assemblies provided to make microsatellite genotype calls, instead each microsatellite was re-built using the raw data and our verified algorithms [11]. The raw sequencing reads obtained for this study through NCBI SRA were downloaded, decrypted, and decompressed using software by NCBI SRA. Then they were filtered based on the quality score requirements set forth by the 1000 Genomes Project [30].

### Microsatellite-based genotyping

Quality filtered reads from The Cancer Genome Atlas [6], were aligned to the human reference genome (NCBI36/hg18) using BWA [31]. Our microsatellite-based genotyping uses non-repetitive flanking sequences to ensure reliable mapping and alignment at microsatellite loci by filtering out all microsatellite-containing reads that do not completely span the repeat as well as provide additional unique flanking sequence on both sides [10]. We then use the unique flanking sequence along with a small portion of the repeat for local alignment of the read to the correct genomic locus. We perform this same procedure on those reads that were not aligned to the reference by BWA, obtaining additional coverage at some loci. Only loci with a coverage of at least 15x in a given sample (healthy or cancer genomes) are considered “callable” and genotyped. See supplemental methods for additional details.

### Modal genotype determination

We compiled the genotypes from all the 1kGP-EUF samples for each microsatellite locus. The genotype supported by the highest number of samples was determined to be the modal genotype. In cases where more than one genotype was equally represented, the genotype listed first in our compiled set was used consistently as the modal genotype.

### Computing statistics for each microsatellite locus

2 × 2 tables were created for each locus for the 1kGP-F normals and the OV germline samples that were called in at least 10 samples in each set: 1kGP-EUF with modal/non-modal genotypes by OV germline with modal/non-modal genotypes. An R script computed the *p*-value for each locus using the two sided fisher.test function. The Benjamini-Hochberg cut-off was selected as 0.01% (FDR < 1/3750 (total number of loci with *p*-value < 1)) to make it unlikely that any locus is a false positive from our data set. 60 loci passed the FDR and were considered to be informative in distinguishing the healthy EUF from the cancer samples. Relative risk for each locus was computed as the percent of individuals with the non-modal genotype from the cancer set divided by the percent of individuals with the non-modal genotype in the normal set.

### ENCODE, etc

ENCODE and related data for the 60 informative microsatellite loci was obtained from the UCSC Genome Browser [32, 33].

### Calculating the risk classifier

Using the 60 loci that significantly differentiated OV genomes from healthy genomes, we plotted an ROC curve for the sensitivity and specificity spectrum and identified the point of inflection as the cut off for identifying an exome as ‘cancer-like’. We then evaluated each exome at the 60 informative OV loci. Any individual exome in which fewer than 10% of the informative loci was genotyped was not included in the subsequent analyses.

### Ontology

GO enrichment analysis of genes associated with the 60 signature loci was performed using DAVID [34, 35] functional annotation tools (*p* < 0.1), Genedecks [36] and GSEA [37]. Pathway enrichment was performed using Panther [38].
